# Timing of Neuropeptide Coupling Determines Synchrony and Entrainment in the Mammalian Circadian Clock

**DOI:** 10.1371/journal.pcbi.1003565

**Published:** 2014-04-17

**Authors:** Bharath Ananthasubramaniam, Erik D. Herzog, Hanspeter Herzel

**Affiliations:** 1 Institute for Theoretical Biology, Charité and Humboldt University Berlin, Berlin, Germany; 2 Department of Biology, Washington University in St. Louis, St. Louis, Missouri, United States of America; Indiana University, United States of America

## Abstract

Robust synchronization is a critical feature of several systems including the mammalian circadian clock. The master circadian clock in mammals consists of about 20000 ‘sloppy’ neuronal oscillators within the hypothalamus that keep robust time by synchronization driven by inter-neuronal coupling. The complete understanding of this synchronization in the mammalian circadian clock and the mechanisms underlying it remain an open question. Experiments and computational studies have shown that coupling individual oscillators can achieve robust synchrony, despite heterogeneity and different network topologies. But, much less is known regarding the mechanisms and circuits involved in achieving this coupling, due to both system complexity and experimental limitations. Here, we computationally study the coupling mediated by the primary coupling neuropeptide, vasoactive intestinal peptide (VIP) and its canonical receptor, VPAC2R, using the transcriptional elements and generic mode of VIP-VPAC2R signaling. We find that synchrony is only possible if VIP (an inducer of *Per* expression) is released in-phase with activators of *Per* expression. Moreover, anti-phasic VIP release suppresses coherent rhythms by moving the network into a desynchronous state. Importantly, experimentally observed rhythms in VPAC2R have little effect on network synchronization, but can improve the amplitude of the SCN network rhythms while narrowing the network entrainment range. We further show that these findings are valid across several computational network models. Thus, we identified a general design principle to achieve robust synchronization: An activating coupling agent, such as VIP, must act in-phase with the activity of core-clock promoters. More generally, the phase of coupling is as critical as the strength of coupling from the viewpoint of synchrony and entrainment.

## Author Summary

Synchronization among multiple oscillators is a common theme in many biological and engineered systems. Here, we look at its use by the mammalian biological clock to keep accurate time. Through biochemical interactions among a network of inaccurate neuron clocks, a strong precise clock is produced. Although we are gradually learning more about these biochemical interactions, the details still remain largely unclear. Studies, both computational and experimental, have shown that the strength of the rhythmic interaction critically decides if a system can synchronize, i.e., the interactions must be strong enough. In this work, we show that the rhythmic interaction between these neuronal clocks must be timed correctly (in the right phase) in addition to being strong enough to synchronize the network. Activating (repressing) interactions must coincide with other activators (repressors) in each neuron to achieve synchrony. Since this principle imposes certain properties on synchronizing interactions, these properties can be used to identify and understand novel interaction mechanisms. Further, these principles are applicable to interactions between cellular oscillators in other tissues and organisms.

## Introduction

Organisms evolved an internal biological timekeeper or ‘clock’ to temporally organize and regulate their physiological processes to best cope with a fundamentally periodic natural environment. In mice, the model system in mammals, this master biological clock consists of 20000 neurons within the suprachiasmatic nucleus (SCN) in the hypothalamus. Each neuron sustains oscillations in the expression of ‘clock genes’ and firing that are noisy and variable. Inter-neuronal coupling [1], [2] lends both precision [3] to the clock via synchronization and robustness to the clock against certain perturbations of the individual neuronal oscillators [4]. Coupling between neurons is mediated by synapses, gap junctions, and neuropeptides, such as vasoactive intestinal peptide (VIP) [5]. Synchronization produced by neuropeptide-based coupling is the central focus of this work.

VIP is one of the most important and well-studied coupling agents responsible for synchrony in the SCN [1], [6], [7] along with its canonical receptor VPAC2R [8]. In mice, the abolition of VIP-based signaling by knockout of VPAC2R or VIP, or both, leads to weak rhythms or arrythmicity in behavior, clock protein oscillations, and firing rate in SCN explants [1]. The rhythmicity in a VIP knockout SCN explant can be restored by grafting a normal donor (wild-type) SCN via only paracrine VIP signaling [9]. The down-stream targets of VIP binding to VPAC2R acting via G-protein coupled receptor pathways involving the second-messengers cAMP and 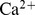 are activators of the period clock genes (*Per1*,*Per2*) [10], and VIP pulses can entrain SCN explants much like periodic light stimuli in mice [11]. VPAC2R has been also shown to be intracellularly expressed in a circadian manner, with the expression peaking in the early morning [12]. While the circadian expression/release of VIP is expected, *in vivo* VIP levels have not been conclusively measured [13]–[18].

A complete understanding of this critical synchronization phenomenon in the SCN remains an open question. In experimental and computational studies, coupling individual oscillators can achieve this robust synchrony, despite heterogeneity and different network connectivities. But, much less is known regarding the mechanisms and circuits involved in achieving this coupling, both due to system complexity and experimental limitations (as described above for VIP-VPAC2R coupling). Limiting the scope of possible coupling mechanisms that can achieve synchronization would have significant scientific value.

Ueda et al. [19] and Korenčič et al. [20] identified the interactions between essential clock components (termed ‘core oscillator’) using only conserved transcriptional elements and phase relationships between core components, and thus outlined design principles for robust transcriptional oscillations. Similarly, in this work, we identify design principles behind VIP-VPAC2R-mediated coupling for robust synchrony, given the transcriptional elements and generic mode of VIP-VPAC2R coupling (assuming VIP-VPAC2R is the primary means of coupling). In particular, we computationally test the hypothesis that the phase of either VIP or VPAC2R, or both, relative to the core clock determines SCN synchrony and affects SCN properties, such as amplitude, period and entrainment range.

We find that synchrony is only possible if VIP (an inducer of *Per*) acts in-phase with the transcriptional activity of *Per* promoters. Moreover, anti-phasic VIP action suppresses the network rhythms by moving the network into a desynchronous state, without completely suppressing individual oscillators. The modulation of afferent VIP signaling by phase-dependent VPAC2R expression [12] primarily improves the amplitude of SCN network rhythms and narrows the network entrainment range and has little effect on the ability of the network to synchronize. Finally, we have identified that an activating coupling agent, such as VIP, must follow a coupling-in-phase-with-promoters design principle to achieve robust synchrony, as an extension of the work of Ueda et al. [19]. Our findings are also applicable across several proposed models of the SCN network.

Early computational models of the SCN focused on the fundamental biochemical mechanisms of the transcriptional-translational feedback loop (TTFL) that produces oscillations in the SCN [21], [22]. With the availability of real-time clock reporters and siRNA technology, detailed quantitative models of single SCN neuronal oscillators have been proposed [4], [20], [23]–[25]. Simultaneously, studies have considered properties of the SCN network vital to producing sustained and high precision oscillations: the role of strength of coupling [26]–[28], the role of network connectivity [29], [30], composition of damped and sustained neuronal oscillators [31], and modes of coupling [32]. These studies on coupling assumed a neuropeptide under clock control of sufficiently large amplitude to achieve synchrony. They focused, therefore, on the strength of coupling rather than on the effects of changes in the timing of coupling.

The theory of coupled oscillators describes the role of phase of coupling in determining synchronous and asynchronous states of an oscillator network under the assumption of weak coupling between oscillators (see [33] and references therein). In the circadian realm, Indic et al. [34] show that an altered phase of coupling is necessary to split synchronized SCN oscillations into two anti-phase subgroups and thus explain the splitting behavior in hamsters using a theoretical formulation similar to ours. Ueda et al. [35] use a principle that synchronizing factors released during the day and night must have similar phase response curves to light- and dark-pulses, respectively, to identify potential coupling mechanisms in *Drosophila*. The work of Ueda et al. [35] is consistent with our theory and also suggests a role for the phase of these factors in synchrony. Our work is, to our knowledge, the first systematic investigation into the role of the phase (or timing) of neuropeptide coupling in determining properties of the SCN network, including synchrony and entrainment. We show that the phase of coupling is as important as and complements the amplitude of coupling in determining synchrony and entrainment across multiple models. Moreover, we explicitly consider the circadian control of the ligand (VIP) and receptor (VPAC2R) and highlight the asymmetry in the roles played by VIP and VPAC2R.

## Results

We wish to study the effects of both the timing of VIP release and VPAC2R expression on SCN synchrony and entrainment. Therefore, we first consider the timing of VIP release with constant (constitutive) VPAC2R expression and later incorporate periodic VPAC2R expression at different phases. Intuitively, periodic VIP release is necessary for the model to synchronize as the VIP phase informs neighbors of the phase of the releasing neuron's clock.

**Figure 1:**
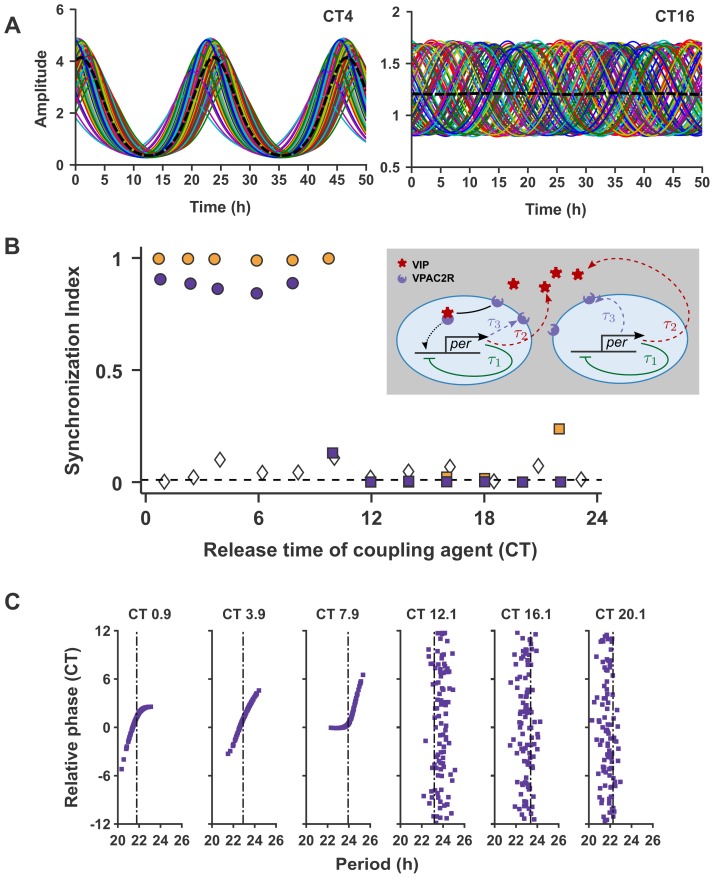
Simulations with constant VPAC2R expression. (A) *Per* gene expression in individual neurons in two networks with different times of neuropeptide release: (*left*) VIP release at CT4 leads to synchrony and (*right*) VIP release at CT16 leads to desynchrony; (B) The SI quantifying network synchrony at different VIP release phases for two different spreads of periods in the network: nominal (purple) and low (yellow). Circles represent VIP release phases when network synchrony is achieved (when all neurons have the same period), while squares indicate incomplete network synchrony (when not all neurons have the same period). The diamonds are SI for an uncoupled network. (*Inset*) Schematic of the VIP-VPAC2R-coupled SCN neuron model. The timing of VIP release and VPAC2R expression are controlled by the delays 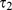 and 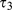, while 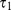 is the delay in the negative feedback of the transcriptional-translational feedback loop (TTFL); (C) The intrinsic period of neurons versus their peak phases relative to a reference neuron (for every other release time in (B)). Positive (negative) relative phase imply a peak after (before) reference neuron peak. The dash-dot line is the period of the network rhythm with corresponding timing of neuropeptide release.

We simulate the network model in Figure 1B (inset) consisting of 100 neurons with a fixed amplitude, but with intrinsic (uncoupled) periods drawn from a normal distribution with a mean of 23.7 h and nominal period spread (standard deviation) of 0.6 h, in accordance with [36]. We initially use an all-to-all network connectivity with no autocrine interactions, but we later evaluate other network connectivities as well. We use certain delays (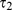 and 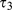) in the model to control the phase of VIP release and VPAC2R expression, respectively (see Figure 1B (inset)). The *phase* of a periodic signal is the timing of the peak of the signal. Following chronobiology conventions, the phase over one cycle spans circadian time (CT) 0 to 24 and the peak in *Per* gene expression is the reference phase CT6. The time of day between CT0-12 and CT12-24 are termed *subjective day* and *subjective night*, respectively. The effect of the external light-dark environment is simplified as a sinusoidal VIP signal superimposed on the network in order to qualitatively understand the entrainment properties of the system. Detailed cellular modeling of the complex effect of light on VIP and the endogenous neuronal oscillator is beyond the scope of this work.

We quantify synchrony in the network using different metrics. Period synchrony is required for network synchrony. So, when the entire network oscillates with same period (in practice, if the variance of periods is below a threshold), then we test phase synchrony in the network using the Synchronization Index (SI). The SI or Kuramoto order parameter [37] measures the phase alignment of a period synchronized network and takes values between zero and one. The SI is zero, when the phases of neurons are evenly spread between CT0 and CT24, and one, when all neurons are phase aligned. Network rhythm refers to *Per* oscillations averaged over the entire population of neurons. We can thus assign an amplitude and period to this network rhythm. It is worth noting that the amplitude or period of the network rhythm is different from mean of the amplitudes or periods of individual oscillators in the network.

### Only VIP release in the early subjective day leads to synchrony

We first study the effect of the timing of VIP release alone on network synchrony and entrainment by setting VPAC2R expression to be constant. In addition to the VIP release time, we also varied the spread of intrinsic periods in the network in order to test the robustness of our results. The results are summarized in Figure 1. Time courses of the computational SCN reveal either synchrony (with VIP release at CT4) or desynchrony (at CT16) (Figure 1A). Synchrony is only achieved with VIP release in a small window (henceforth called the region of synchrony (ROS)) within one circadian cycle (Figure 1B). The network always synchronizes for VIP release in the early subjective day (CT0-CT6), the time of peak intrinsic *Per* production, irrespective of the heterogeneity (period spread) of the network.

Moreover, the system exhibits sharp transitions between regions of synchrony and desynchrony. This reflects a phase transition of the network into a synchronous state, a well-studied phenomenon in classical coupled oscillator theory [38]. This ‘cooperative’ process is nucleated by a small cluster of synchronized neurons that progressively recruit neighbors until the entire network is in synchrony. In particular, synchrony is attained when coupling-induced gene expression and intrinsic gene production in each neuron act synergistically, i.e., coupling and intrinsic gene production are in-phase (for VIP release at CT4, Figure S1A). However, for VIP release at CT16, when the network does not synchronize, the coupling-induced expression and intrinsic production act antagonistically in each neuron (Figure S1A).

The width of the ROS is dependent on the spread of intrinsic (uncoupled) periods of neurons in the network and the strength of coupling between neurons. The ROS is broadened (from CT0-CT8 to CT0-CT10) as the period spread in the network is decreased from its nominal value. Increasing the strength of coupling between neurons by over-expressing either VIP or VPAC2R broadens the ROS and can thus compensate for increases in period spread of the network (Figure S1B). While the network achieves perfect phase synchrony at low period spread with an SI of one, at the nominal spread, the phases of individual neurons are not perfectly aligned (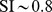) (see Figure 1B). The snapshot of rhythms in individual oscillators in the synchronized network shows that neurons with nominal period spread are arranged in a linear phase pattern (Figure 1C). Within this pattern, some neurons peak earlier (phase advanced) than the network rhythm, while others peak later (phase delayed). Neurons with intrinsic period longer than the mean period are phase advanced and neurons with shorter intrinsic period are phase delayed (Figure 1C). The width of the linear phase ordering is significantly compressed as the period spread of the network is decreased (Figure S1C).

Outside the ROS, while the neurons continue to oscillate (Figure 1A), the phases of the neurons are dispersed (Figure 1C). Although the neurons continue to interact, their ordering strongly resembles an uncoupled network of neurons as measured by the SI (Figure 1B). Despite the strong coupling signal between neurons in the network, the coupling maintains the system in a ‘uncoupled’-like state and prevents synchrony. An almost perfect positive correlation between individual neuron periods in the desynchronized network and their intrinsic periods further supports this view (not shown). Thus, the coupling might be viewed as forcing desynchrony in the neuronal network.

The period of the *Per* gene network rhythms is close to the average intrinsic period of individual neurons in the network (Figures S1D and S1F). The period of the network rhythm is modulated by the phase of VIP release within the ROS. The amplitude of the network rhythms is boosted by synchrony within the ROS as measured by both absolute amplitude and amplitude relative to the mean *Per* expression (Figures S1E and S1G). While individual neuron amplitudes without VIP-VPAC2R signaling is 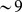, coupling reduces individual neuron amplitudes irrespective of the VIP release phase. Individual neurons in the coupled network have smaller amplitudes outside the ROS as compared to within the ROS. In other words, there is a significantly higher amplitude reduction (penalty) in desynchrony than in synchrony.

The network entrains to sufficiently-strong external light-dark cycles (Zeitgeber) both within the ROS (at CT4) and outside the ROS (at CT16) (Figure S2A). Thus, the phase of VIP release does not affect the ability of the network to entrain to a Zeitgeber. However, the network shows different entrainment characteristics with VIP release at CT4 and at CT16. A synchronized SCN network (VIP release at CT4) behaves as a ‘strong’ oscillator that shows a narrow entrainment range and a phase angle of entrainment highly sensitive to the Zeitgeber period (see [39], [40] for definition of ‘strong’ and ‘weak’ oscillators). On the other hand, an SCN under desynchrony (VIP release at CT16) behaves as a ‘weak’ oscillator that exhibits a wide entrainment range and a phase angle of entrainment less sensitive to Zeitgeber period.

### Circadian VPAC2R expression in the early subjective day boosts circadian rhythm amplitude and narrows the network entrainment range

**Figure 2:**
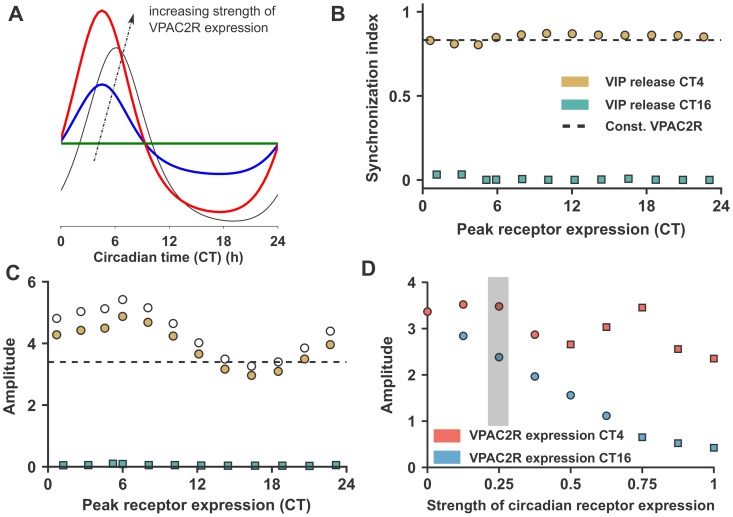
Simulations with circadian VPAC2R expression. (A) Variation of VPAC2R expression profile for increasing fraction of expression under circadian control (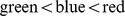). The shape of VPAC2R expression is the same as the *Per* expression (black); (B) Synchrony measured with SI for different peak phases of VPAC2R expression at two different VIP release phases: CT4 within the ROS and CT16 outside the ROS. The SI for constant VPAC2R expression with VIP release at CT4 is also shown for comparison; (C) The network rhythm amplitude with VIP release at CT4 and CT16 following the same color scheme as (B). The open circles are the mean of individual neuron amplitudes in the network with VIP release at CT4. The dashed line is the network amplitude for VIP release at CT4 with constant VPAC2R expression for reference. (D) Variation in network amplitude with different strengths of circadian VPAC2R receptor expression for VPAC2R expression at CT 4 and CT16 and VIP release at CT4 (corresponding to yellow plot in panel (C)). The shaded region is the strength of circadian VPAC2R expression used in plots (B) and (C). (In all panels, circles are used when period synchrony is achieved and squares when incomplete synchrony is reached).

We now further introduce circadian VPAC2R expression into the model. We study both the effect of the timing of VPAC2R expression and the strength of circadian modulation of VPAC2R when VIP is released at either CT4 or CT16. VPAC2R expression is under *Per* TTFL control and its strength is varied, while keeping the mean VPAC2R expression a constant, as shown in Figure 2A. Surprisingly, the ability of the network to synchronize is unaffected by the peak phase of VPAC2R expression (Figure 2B). As before, the SCN network can synchronize only with VIP release in the early subjective day (CT4), but not in the subjective night (CT16).

The phase of VPAC2R expression affects, nevertheless, the amplitude of both individual oscillators in the network and the network rhythm (Figure 2C). VPAC2R peak expression in the early subjective day (coinciding with the ROS for VIP release) significantly improves the amplitude of individual oscillators and the network rhythm relative to constant VPAC2R expression. This boost in the network amplitude is caused by an increase in individual rhythm amplitude, since the synchronization index remains unchanged for different VPAC2R phases. VPAC2R expression in the subjective night suppresses the network rhythm and individual amplitudes. We also observed modulation of the network period by the phase of VPAC2R expression (Figure S3A).

Next, we vary the strength of VPAC2R modulation between 0, meaning constant VPAC2R expression, and 1, meaning maximal circadian VPAC2R expression with no basal component (see Figure 2A). The amplitude of the network rhythm initially increases and then starts to decrease with increased strength of VPAC2R modulation for VPAC2R expression peak at CT4 (Figure 2D). Recall that VPAC2R expression at CT4 enhances network rhythm amplitude by boosting individual amplitudes (Figure 2C). On the other hand, with VPAC2R expression at CT16, where VPAC2R modulation reduces network amplitudes, increasing the strength of VPAC2R expression reduces amplitudes even further. However, while increasing strength of VPAC2R expression at CT16 reduces amplitudes, it results in period synchrony over a larger range of VPAC2R expression strengths than with VPAC2R expression at CT4. Similarly, the network period increases and decreases with changes in the strength of VPAC2R expression with VPAC2R peak at CT4 and CT16, respectively (Figure S3B).

The entrainment properties of the network are also modulated by the phase of VPAC2R expression irrespective of the phase of VIP release (Figure S2B). Peak VPAC2R expression in the subjective night (at CT16) leads to an wider entrainment range relative to constitutive VPAC2R expression (Figure S2A) in both a synchronized (VIP release at CT4) and desynchronized (VIP release at CT16) SCN network. In other words, VPAC2R expression at CT16 makes the network behave as a ‘weaker’ oscillator than with constitutive VPAC2R expression [39]. On the other hand, while peak VPAC2R expression in the early subjective day (CT4) results in a much narrower entrainment range or a ‘stronger’ oscillator, peak VPAC2R expression at an intermediate phase (CT10) leads to an intermediate network entrainment range. Thus, VPAC2R expression phase can smoothly tune the entrainment range of the SCN network and the network's qualitative behavior as a ‘strong’ or ‘weak’ oscillator.

### The effect of the phase of VIP release and VPAC2R expression is independent of model and network properties

**Figure 3:**
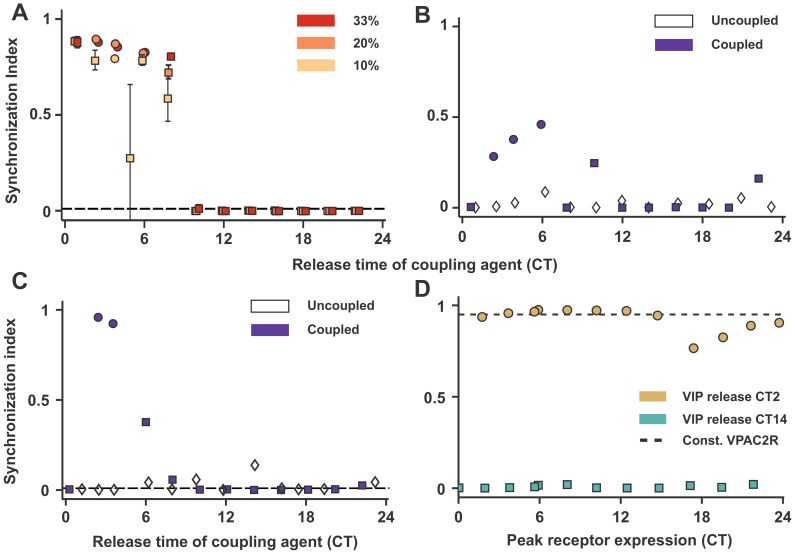
Effect of network topology and neuronal oscillator model. (A) Synchrony in a network with random connections for different degrees of connectivity (33%, 20%,10%) with the model defined in this work (Figure 1B Inset), each averaged over 5 different network realizations; (B) Synchrony measured by SI with neurons arranged in a ring with each neuron connected to 20 neighbors on either side on the ring (a local topology with 20% connectivity); (C) Synchrony measured with SI for the Goodwin-based neuron model of [26] with different times of VIP release (compare against Figure 1D); (D) Synchrony measured with SI in the Goodwin-based neuron model from [26] for different peak phases of VPAC2R expression at two different VIP release phases: CT2 within the ROS and CT14 outside the ROS (compare with Figure 2B). The SI for constant VPAC2R expression with VIP release at CT2 is also shown for comparison. (In all panels, circles are used when period synchrony is achieved and squares when incomplete synchrony is reached).

Finally, we test the robustness of our previous results on the network topology in the proposed model and the choice of model. We studied first a randomized network topology, since in the SCN only 10% of SCN cells express VIP [41] and only 30% of VIP cells express VPAC2R [42] making the network connectivity very heterogeneous. Replacing the all-to-all network connection with a random network topology or a local ring topology has little effect on the qualitative conclusions on synchrony and the timing of VIP release (Figures 3A and 3B). The change in topology and percentage connectivity of each neuron only alters the width of the ROS. Our conclusions regarding forced desynchrony, when the activating coupling agent is not in-phase with promoter of *Per* production, also remain unchanged.

The qualitative observation that VIP release only in the early subjective day leads to synchrony and a lack of effect of the peak phase of VPAC2R expression on network synchrony appears to be robust to the choice of model as well. In the Goodwin-like model of Gonze et al. [26] defined in (7), the ROS for constitutive VPAC2R expression is restricted to a small window between CT2-4 (Figure 3C). As before, the poor synchrony in the subjective night is driven by desynchrony in the neurons as evidenced by robust individual neuron oscillation amplitudes, but very small network rhythm amplitudes (Figure S4A). As before, with oscillatory VPAC2R expression, the network remains in a state of synchrony (VIP release at CT2) or desynchrony (VIP release CT14) irrespective of the peak phase of VPAC2R expression (Figure 3D). However, as before, the phase of VPAC2R expression modulates the network amplitude and period (Figure S4B).

One feature shared by both our DDE model and the Goodwin-like Gonze model is that VIP-VPAC2R coupling acts independently of the endogenous *Per* gene transcription according to an ‘OR’-logic gate (discussed in the Models section). While this is consistent with experimental data on the *Per* promoter [43], several prior models [26], [28], [29], [31] assume a dependent ‘AND’-logic gate for the coupling-driven and endogenous gene transcription. We verified using a variant of our DDE model (5) that our results were insensitive to the precise mechanism of coupling-driven and endogenous *Per* gene transcription (Figure S4C).

## Discussion

Our first key result is that VIP-based coupling must have a peak phase in the early subjective day to ensure synchronization in the SCN network. In showing this, we assumed that some combination of VIP production and release is under circadian control resulting in periodic VIP-based coupling (ignoring effects of the light environment). The experimental evidence regarding this assumption is mixed and sometimes inconsistent in mice and rats, two closely-related model species. In rats, overwhelming evidence suggests that both VIP mRNA expression and protein levels do not oscillate in constant darkness (DD condition) and oscillate only under light-dark (LD) conditions [15], [17], [44], [45]. However, VIP released into the extracellular medium in cultured rat SCN is circadian [18]. In mice, Dardente et al. [13] and Laemle et al. [16] show that VIP mRNA and protein levels in the SCN are circadian even under DD conditions. Although the circadian nature of VIP release is debated, we can study its effects via computational modeling.

We identified an important design principle for coupling in the SCN: for activating coupling agents, the inducing activity of the coupling agent must be in-phase with the endogenous activity of the target gene's promoters. This result is aided by our choice of a minimalist delay-based model of the SCN, and the generality of this principle across other choices of SCN model and network connectivity further strengthens our assertion. In fact, this principle is a generalization of prior work on the phasing of transcriptional regulation in single oscillators [19], [20] to a network of oscillators. Ueda et al. [19] and Korenčič et al. [20] both show that activating transcription factors should be in-phase, and suppressing transcriptions factors must be anti-phase with the activating transcription factors to achieve large amplitude. Clearly, neurons with improved amplitude from optimal inter-neuronal coupling can better influence and align wayward neurons back into a stable relationship. We also expect therefore that coupling agents that suppress core clock transcription or enhance degradation to follow the anti-phase with intrinsic gene production design principle, although we do not have a biological example for this.

Antiphase VIP action results in desynchrony among robustly oscillating neurons resembling adult SCN in 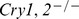 mice [46]. Moreover, 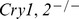 SCN explants from neonates are synchronous and lose synchrony only during development. Our theory on timing of coupling suggests an explanation for this effect, since the phase of VIP action has been measured to change by several hours with age [44]. Recent work on another coupling factor, GABA, has shown that it works against synchrony under steady-state conditions [47], but acts to resynchronize the SCN under anti-phase configurations [48]. These observations are consistent with the hypothesis that GABA signaling endogenously occurs at the ‘incorrect phase’ (out-of-phase with repressors, since GABA is an inhibitory neuropeptide). Thus, GABA normally reduces synchrony among neurons, and only when neurons of the dorsal and ventral SCN are forced out-of-phase under extreme photoperiods [48], GABA behaves as a synchronizing neuropeptide. This role reversal of GABA with the phase configuration of the SCN is similar to the change in behavior of VIP from a synchronizer to a desynchronizer with overexpression of the neuropeptide [49] and supports our view that the phase and strength are complementary features of coupling.

Entrainment of the network to an external Zeitgeber revealed differences in its behavior under synchronizing and desynchronizing VIP action. A synchronized network behaves as a ‘strong’ oscillator, while a desynchronized network behaves as a ‘weak’ oscillator. Comparing a coupled SCN network and uncoupled lung tissue, Abraham et al. [39] show by measuring entrainment ranges that the strength of coupling determines the ‘strong’ or ‘weak’ nature of oscillators. Our results reveal that even for the same strength of coupling, the phase of coupling determines the entrainment behavior of the SCN oscillator. This complementarity between coupling strength and phase also applies to recent observations that weakened coupling by means of vasopressin receptor knockouts speeds up jet-lag shifts in mice [50]. Simulations of 8 h jet-lag advances and 8 h delays showed faster re-entrainment with anti-phase VIP action (i.e., in a desynchronized SCN) (Figure S2C). These results are also consistent with the recent finding that desynchronizing the network with VIP prior to a jet-lag protocol leads to faster re-entrainment after the jet-lag shift [49].

Another general feature of this coupling is the asymmetric effect of circadian control of the neuropeptide and its receptor on synchrony in the network. Synchrony in a network requires exchange of phase information between the constituent oscillators. Circadian release of neuropeptide informs the network of the phase of the releasing neuron aiding consensus in-phase and ultimately synchrony. However, the phase of the receptor merely modulates the effect of the rest of the network on the neuron. Thus, circadian neuropeptide release is globally informative, while circadian receptor expression is only locally informative.

Next, we assemble available literature data on the timing of different aspects of VIP-VPAC2R signaling. Laemle et al. [16] show VIP immunoreactivity within SCN neurons peaking in the early subjective day and late subjective night entirely consistent with our prediction. Dardente et al. [13] estimate peak VIP mRNA expression at around CT18 allowing VIP release to occur in the early subjective day after delays in translation and extracellular release. While Shinohara et al. [18] show VIP release in multiple rat SCN explants in a range between CT16-CT24, attribution of CTs is made difficult by significantly shortened period (approx. 21 h) of VIP oscillations. Moreover, these measurements come from neonatal SCNs and there is some evidence that VIP mRNA phases change by up to 12 hours during development [44]. The available data on VIP timing are thus mostly consistent with our prediction of VIP release in the early subjective day.

Information on second-messengers and transcription factors in the G-protein coupled receptor-mediated VIP-VPAC2R signaling to the core circadian clock [7] can also be used to test our predictions indirectly. Brancaccio et al. [51] and Enoki et al. [52] both show using real-time reporters that intracellular 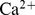 oscillates and peaks at CT6-7. Moreover, cAMP, the other important second-messenger, also peaks in the early to mid subjective day (CT6) as reported by O'Neill et al. [53]. Finally, VIP-VPAC2R signaling converges on the core circadian clock through the transcription factor CREB. CREB-mediated activation of *Per* gene expression occurs in the mid-subjective day (CT6) [54]. Although this evidence reinforces our claim that coupling must occur within a certain circadian time window in the subjective day, the particular CT of action is likely to be determined by other factors including entrainment, period control and SCN heterogeneity.

The most direct measurements of VPAC2R expression under DD conditions comes from An et al. [12] showing peak bioluminescent staining of SCN slices at subjective dawn (CT0). Since we expect synchrony with robust amplitude with VIP in-phase with VPAC2R in the early subjective day, the study reinforces both our claims regarding phase of VIP release and VPAC2R expression. Moreover, VPAC2R mRNA expression is found to either peak around dawn (ZT0) [15] under LD conditions, or be circadian, but biphasic, in DD and LD conditions [55]. However, recent work on post-transcriptional control [56] has shown that having circadian mRNA expression is no guarantee of circadian protein levels and vice versa. Circadian VPAC2R expression did not have a significant effect on synchrony in our model, but could tune the amplitude and period of the network. We also observed that the phase of circadian VPAC2R alters the entrainment behavior of the network by modulating the network entrainment range. That VPAC2R expression in the early subjective day narrows the entrainment range is expected, since under similar VPAC2R expression phase we observe increased network amplitude. As noted in [39], changes in network amplitude and period indeed affect the entrainment properties of the network oscillator. Although we identify several critical network properties modulated by circadian VPAC2R expression, we cannot resolve the specific role of the circadian VPAC2R expression that is observed [12].

Evidence in the literature cited above support our claims, but our predictions could be more directly tested. An experiment that significantly alters the phase of VIP release in an SCN explant would be expected to desynchronize the individual rhythms and damp out network rhythms. It would, for example, be interesting to test the effects of putting the *Vip* gene under the control of the *Bmal1* promoter. This would, in principle, put VIP production in anti-phase to its normal physiological release time. A more easily-accessible phenotype of alterations in the phase of VIP-VPAC2R coupling is the period of an explant or behavioral rhythms. The period of network rhythms is affected by both phase of VIP release and VPAC2R expression in our model (Figures S1C, S2E and S3A). Moreover, the phase angle of entrainment would also change with the change in period. These experiments could follow the approach of previous studies on VIP and VPAC2R signaling [6], [42].

While we focused on the VIP-VPAC2R coupling mechanism, our results are equally applicable to other coupling neuropeptides and their receptors, such as vasopressin (AVP) and its receptor (AVPR) or gastrin-releasing peptide (GRP) and its receptor in mammals. Our work also raises interesting possibilities for multiple coupling neuropeptides with different strengths and phases operating synergistically to synchronize and entrain the SCN under different physiological and environmental conditions, such as with GABA and VIP observed in [48]. We did not, however, take into account the heterogeneity in the expression of these different neuropeptides and their receptors. VIP is mainly expressed in the core region, where oscillators appear to be weak or damped, while AVP is expressed predominantly in the shell with robust oscillations [57]. VIP is expressed in 10% of SCN neurons [41], up to 90% of neurons express the VPAC2R [12], thereby allowing these neurons to respond to VIP signaling. While VPAC2R is expressed in only 30% of VIP cells, it is expressed in 50% of AVP cells [42]. So the core VIP neurons seem to signal a different class of shell AVP neurons. Thus, there is a complex topology of interactions and core clock properties [58] that we have not accounted for. Since our results are based on the interaction of coupling with a core clock mechanism, they are qualitatively independent of these specific neuronal and network properties and could be applied to other functionally-similar neuropeptides in other organisms, such as pigment dispersing factor (PDF) in *Drosophia* [7], [59].

## Models

We use a minimalist computational model of each neuronal oscillator and VIP-VPAC2R-based coupling with the fewest assumptions in order to identify design principles in the SCN. This minimal model course-grains more detailed molecular models of the SCN [4], [20], [23]–[31] by focusing on the underlying qualitative features of circadian neurons relevant to VIP-VPAC2R-mediated coupling. Oscillations in each SCN neuron originate from a transcriptional-translational feedback loop (TTFL) [60], where core clock proteins suppress their own transcription after a time delay due to processes such as translation, post-translational modification and nuclear transport. A periodic signal is defined by its amplitude, period and phase; in practice, the amplitude is the distance between the maximum and minimum, period is time elapsed between successive maxima, and the phase is the timing of a marker, such as the peak, within one period. In such periodic signals, there is a definite relationship between time and phase, and thus, a phase difference is equivalent to the time elapsed between events. This permits phase differences between biochemical processes to be simply described using time delays, using a formulation called delay-differential equation (DDE) models. From a technical viewpoint, a DDE formulation allows the representation of components with different phases using the fewest model parameters. Using this idea, the interaction of 6 core genes in the circadian liver clock was modeled by Korenčič et al. [20].

The TTFL in each neuron is reduced to a single clock gene *Per* that is transcribed and then inhibits its own transcription after a delay. In other words, the TTFL is a negative feedback loop with a delay. This lumped gene is conceived as a combination of two core clock genes, period 1 (*Per1*) and period 2 (*Per2*). These two clock genes are upregulated by VIP [10], and VIP-based coupling causes additional production of the lumped *Per*. The availability of bioluminescence time series from mouse SCN explants *Per1:luc* transgene and PER2::LUC fusion protein made this model choice convenient [61].

The *Per* mRNA 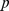 in each neuron is produced at a constant rate, but suppressed after a delay 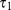 (see first term in rhs) and linearly degraded:
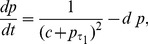
(1)with the notation 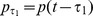. The conditions and choice of parameters 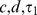 that lead to oscillations with a period of about 24 h have been discussed in [20, Supplementary Information].

While VIP coupling increases *Per* expression [10], the mechanistic effect on *Per* gene regulation in (1) is unclear. On the one hand, coupling-induced expression and intrinsic mRNA production might function like an ‘OR’ logic gate, where one of these two modes of gene expression is sufficient to produce *Per* mRNA [43]. On the other, coupling-induced transcription *in vivo* (outside the promoter constructs of [43]) might function through enhancers that require both modes of expression behaving like an ‘AND’ logic gate. In this work, we use the ‘OR’ logic as it is supported by more evidence. Nevertheless, we also tested the ‘AND’ logic formulation where coupling-driven transcription is also modulated by endogenous gene activation (see Figure S4C).

As described earlier, the biophysical mechanism of *Per* expression by VIP-VPAC2R binding is largely unknown. Exogenous VIP addition appears to produce a transcriptional response much faster than the 24 h time-scale of circadian oscillations (Erik Herzog, personal communication). We thus assume that the VIP-VPAC2R complex immediately and proportionally upregulates *Per* expression:
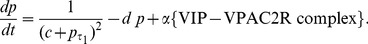
(2)The experimental and theoretical formulations of receptor-ligand binding is discussed in detail in [62]. The simplest model for a ligand (L) binding receptor (R) to form a complex (C), 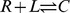, is
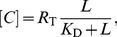
(3)where 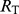 is the total receptor number per cell and 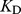 is the dissociation constant for the binding. In biological terms, this model (3) assumes fast binding kinetics and the binding being receptor-limited. It is unknown in the SCN whether VIP or VPAC2R is limiting, and hence, we might also consider the alternate scenario of limiting VIP, which we leave for the future. The coupling upregulates *Per* gene expression in proportion to the complex concentration 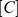 (2).

We consider VIP and VPAC2R to be under core clock control, since the features of VIP release and VPAC2R expression are not well known and beyond the scope of this work. We assume that both VIP and VPAC2R have the same shape as *Per* gene expression. We have checked that the qualitative results presented here are unaffected by this assumption using waveform shaping functions.

The timing of VIP release and VPAC2R expression relative to *Per* expression are varied using time delays 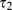 and 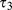, respectively (see Figure 1B (inset)). The SCN neurons release VIP into the common extracellular medium, and thereafter VIP binds to the nearby VPAC2R. The SCN neurons influenced by VIP release from a particular neuron determine the connectivity (outgoing links) of the releasing neuron. We leave open the possibility that a neuron might signal itself (autocrine) in addition to other neurons (paracrine).

The 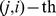 element of the connectivity matrix 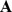 is the contribution of VIP release from neuron 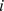 to binding at neuron 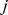. All neurons are fixed to release the same amount of VIP in order to equalize their influence on the network, i.e., columns of 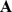 add to unity. We then explore the impact of different topologies on the network by our choice of 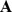.

The total effect of coupling on a neuron is determined by both the total incident VIP and its VPAC2R expression (3). The varying phases of VIP and VPAC2R are then combined using (2) and (3) into
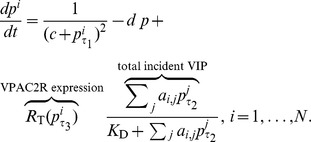
In order to compare the effects of circadian VPAC2R expression against constitutive VPAC2R expression, we normalize the VPAC2R term, such that it is a weighted sum of a constant and circadian expression with a constant mean. The weighting can be altered from 0 to 1 (and values in between) via a parameter 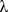 to smoothly transition between constant and fully circadian VPAC2R expressions. We refer to this weighting as the ‘strength of circadian VPAC2R expression’. The model is thus
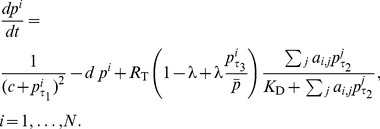
(4)The corresponding set of equations for the ‘AND’ logic gate formulation of *Per* transcription is:
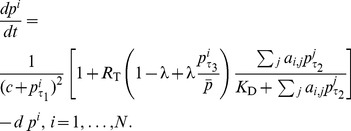
(5)In order to study the entrainment properties of the SCN model, we made a simplifying assumption that external light input contributes to the baseline VIP levels in the extracellular medium of the SCN. Since we focus on synchrony in this work, a detailed exploration of various effects of light on SCN neurons is beyond the scope of this work. We incorporate the effect of light 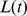 into (4) as:
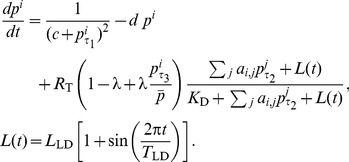
(6)A closely-related coupled oscillator model is the Kuramoto phase model with delayed coupling [63]. The phase-reduced model with coupling defined in [63] shows a similar bistability between asynchronous and synchronous states, and stability of these states can be controlled using the delay (or phase) of coupling between oscillators [64], [65].

We simulate the network model (4) consisting of 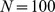 neurons using the dde23 solver in MATLAB [66]. Each neuron's TTFL is parameterized as 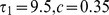 and 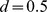 that results in robust oscillations with a 23.7 h period and amplitude of 8.7. The neurons are coupled by VIP-VPAC2R signaling parameterized by the receptor number per cell 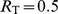 and dissociation constant 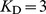. We construct a heterogeneous network with neurons having normally distributed periods (like in [26]), but the same amplitude by scaling both the rate constants and the delays 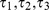. The period spread or standard deviation of the intrinsic neuron periods is used to alter the heterogeneity from low to nominal to high, with nominal being a standard deviation 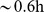 for dispersed SCN neurons [36]. The SCN consists of a mixture of sustained and damped oscillators [58], and damped oscillators aid synchronization of the network [26], [31]. We do not explore these effect here and only study networks of sustained oscillators. For the entrainment simulations, we choose the Zeitgeber strength of 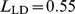 and the Zeitgeber period 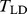 is varied.

We have defined the notion of CT above, but the subtle difference between an individual neuron's and the network's idea of circadian time must be clarified here. One period of *Per* oscillation of a neuron represents 24 circadian hours for that neuron, and VIP release time and VPAC2R expression of this neuron is measured in CT with respect to this neuron's oscillation. This is justified because VIP release and VPAC2R expression are under the control of the respective neuron's TTFL clock. Similarly, the network *Per* oscillation can be used to define a CT for the whole SCN network using the chronobiology conventions outlined earlier. Nevertheless, in this work, we only use CT from the viewpoint of a single neuron, unless otherwise stated.

For a fair comparison of the dynamical properties of the oscillators, such as period and amplitude, between uncoupled and coupled neurons, we compare a single neuron with autocrine signaling against a neuron with only paracrine coupling in the network. These neurons however share parameter values for the TTFL and coupling.

### The Goodwin-based neuron model

We test the robustness of our results (obtained from the DDE formulation (4)) to the choice of model by running simulations using an alternate circadian core model – the one of Gonze et al. [26]. In the modified Gonze et al. model, the circadian oscillator in neuron 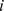 with clock gene mRNA 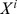, clock protein 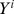 and transcriptional inhibitor 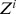 in a network with connectivity 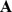 is governed by:
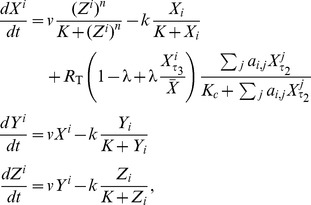
(7)with parameters 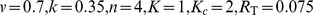. Equation (7) corresponds to the original DDE system in (4) with phase control of both VIP and VPAC2R oscillations by 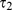 and 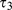 respectively.

## Supporting Information

Figure S1
**Additional plots to constant receptor simulations in** **Figure 1** **.** (A) Comparison on a phase portrait of the rate of instrinsic *Per* production and coupling-induced *Per* gene expression at two circadian times 12 h apart. With VIP release at CT4, instrinsic and coupling-based production are strongly correlated (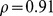), while at CT16, they are strongly anti-correlated (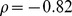); (B) Comparison of synchrony measured using SI in a network with high period spread for nominal coupling strength and increased coupling strength by either over-expression of VIP or VPAC2R (higher than nominal in Figure 1B); (C) *Per* gene expression in the coupled network as neuron phases plotted against the intrinsic period for low period spreads. Compare against the nominal spread plot in Figure 1C; (D) and (F) Comparison of periods of the network rhythm and intrinsic (uncoupled) neuron periods for nominal and low period spreads, respectively; (E) and (G) Amplitude and relative amplitude comparison between mean of individual coupled neurons and network rhythm amplitude corresponding to (D) and (F), respectively. The individual neuron amplitude is 8.7 without any VIP signaling. (In all panels, circles are used when period synchrony is achieved and squares when incomplete synchrony is reached.)(TIF)

Figure S2
**Entrainment of the network to a VIP-based Zeitgeber described by (6).** (A) The phase angle of entrainment of the network with constitutive VPAC2R expression at two phases of VIP release (synchrony at CT4 and desynchrony at CT16) for different Zeitgeber periods (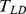) from 19 h to 26 h. The network phase is the timing of the peak of the network rhythm and the Zeitgeber phase is the timing of the peak of the sinusoidal Zeitgeber (see (6)). Period detuning is the difference between the intrinsic period of the network and Zeitgeber period. No phase angle of entrainment is plotted for a particular period detuning if no entrainment is achieved; (B) The phase angle of entrainment versus period detuning like in (A) with oscillatory VPAC2R expression peaks at CT4, 10 and 16; (C) The double-plotted actogram for a simulated jet-lag experiment for 8 h advance (left) and 8 h delay (right) for VIP release causing synchrony and desynchrony and constitutive VPAC2R expression. The network phase and Zeitgeber phase (triangles) are plotted on each day after the jet-lag shift on day 0. On each row, the phases on day 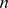 and 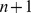 are shown (double-plotted) for easy visualization.(TIF)

Figure S3 **Additional plots to circadian receptor expression simulations in** **Figure 2** **.** (A) Modulation of the network period by the timing of VPAC2R expression with VIP release fixed at CT4; (B) Change in the extent of modulation of the network period by the strength of circadian receptor expression with VIP release at CT4.
(TIF)

Figure S4 **Additional plots to simulations in** **Figure 3** **.** (A) Comparison of network and mean individual neuron amplitudes for the Gonze et al. model [26] for the core oscillator (see (7)) for different phases for VIP release; (B) Modulation of the network amplitude (left) and period (right) by the timing of VPAC2R expression with VIP release fixed at CT2 in the Gonze et al. model; (C) Effect of phase of VIP release in the DDE model in this work for multiplicative (‘AND’ logic gate) activation of *Per* transcription by VIP-VPAC2R coupling as defined in (5). As in Figure 1, the synchronization index, the network time-courses at two different VIP release phases, comparison of network and individual neuron amplitudes and the phase ordering of neurons within the network are shown.
(TIF)
