# Supplementary figures and images for: Timing of Neuropeptide Coupling Determines Synchrony and Entrainment in the Mammalian Circadian Clock
Source: PLoS Comput Biol. 2014 Apr 17;10(4):e1003565. doi: 10.1371/journal.pcbi.1003565 (PMC3990482; doi:10.1371/journal.pcbi.1003565)

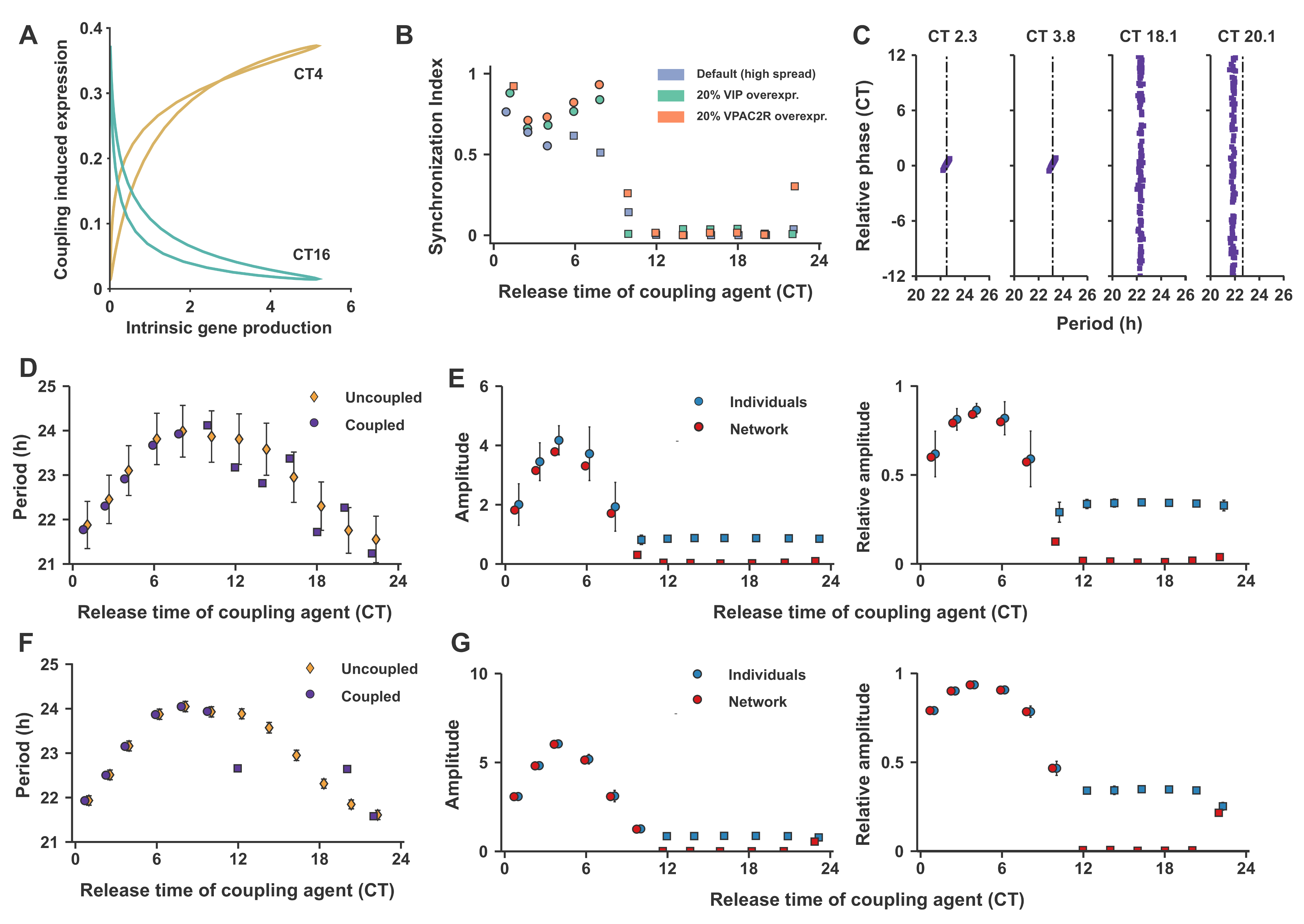

Supplement: Figure S1 — Additional plots to constant receptor simulations in Figure 1 . (A) Comparison on a phase portrait of the rate of instrinsic Per production and coupling-induced Per gene expression at two circadian times 12 h apart. With VIP release at CT4, instrinsic and coupling-based production are strongly correlated (), while at CT16, they are strongly anti-correlated (); (B) Comparison of synchrony measured using SI in a network with high period spread for nominal coupling strength and increased coupling strength by either over-expression of VIP or VPAC2R (higher than nominal in Figure 1B); (C) Per gene expression in the coupled network as neuron phases plotted against the intrinsic period for low period spreads. Compare against the nominal spread plot in Figure 1C; (D) and (F) Comparison of periods of the network rhythm and intrinsic (uncoupled) neuron periods for nominal and low period spreads, respectively; (E) and (G) Amplitude and relative amplitude comparison between mean of individual coupled neurons and network rhythm amplitude corresponding to (D) and (F), respectively. The individual neuron amplitude is 8.7 without any VIP signaling. (In all panels, circles are used when period synchrony is achieved and squares when incomplete synchrony is reached.) (TIF) [file pcbi.1003565.s001.tif]

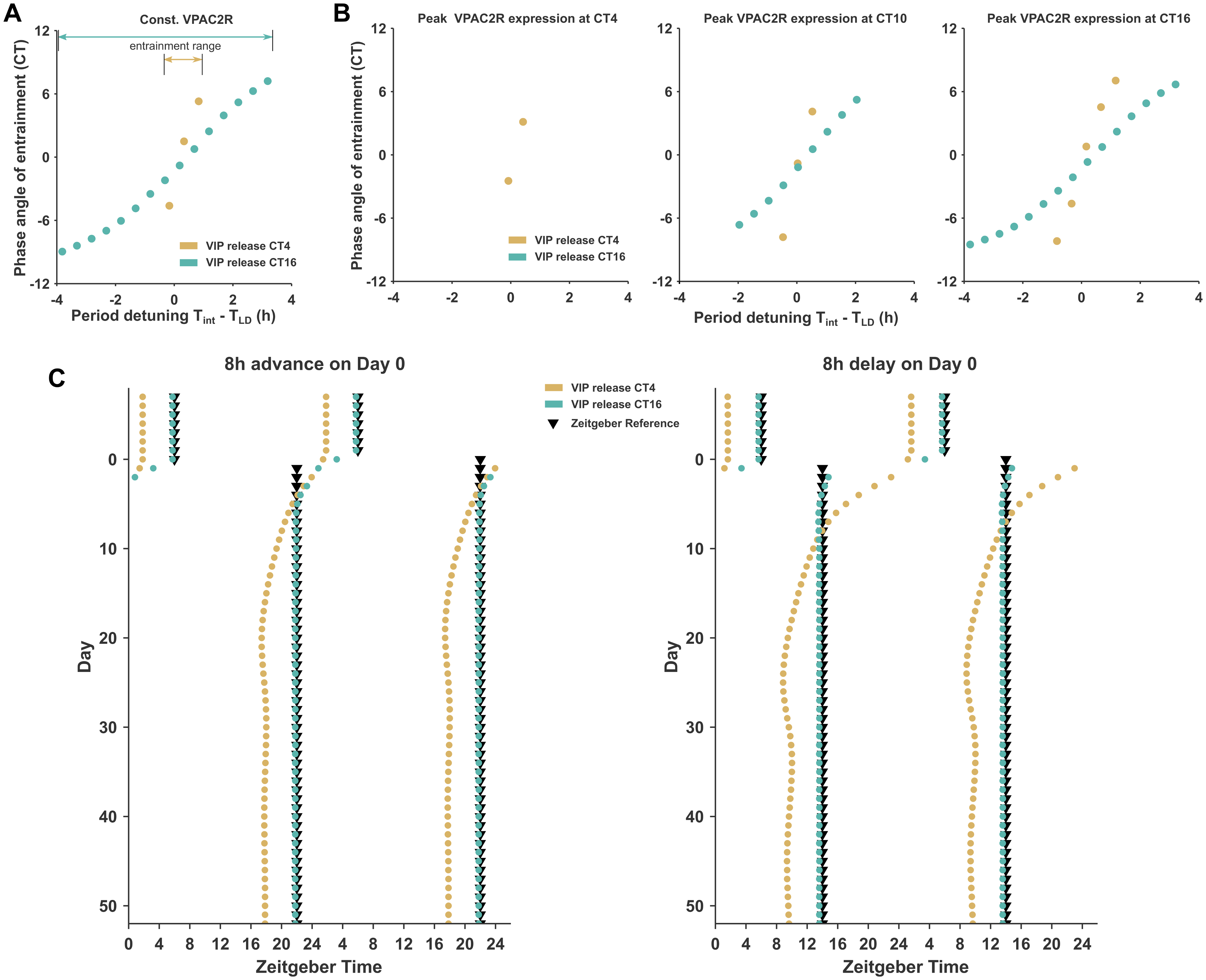

Supplement: Figure S2 — Entrainment of the network to a VIP-based Zeitgeber described by (6). (A) The phase angle of entrainment of the network with constitutive VPAC2R expression at two phases of VIP release (synchrony at CT4 and desynchrony at CT16) for different Zeitgeber periods () from 19 h to 26 h. The network phase is the timing of the peak of the network rhythm and the Zeitgeber phase is the timing of the peak of the sinusoidal Zeitgeber (see (6)). Period detuning is the difference between the intrinsic period of the network and Zeitgeber period. No phase angle of entrainment is plotted for a particular period detuning if no entrainment is achieved; (B) The phase angle of entrainment versus period detuning like in (A) with oscillatory VPAC2R expression peaks at CT4, 10 and 16; (C) The double-plotted actogram for a simulated jet-lag experiment for 8 h advance (left) and 8 h delay (right) for VIP release causing synchrony and desynchrony and constitutive VPAC2R expression. The network phase and Zeitgeber phase (triangles) are plotted on each day after the jet-lag shift on day 0. On each row, the phases on day and are shown (double-plotted) for easy visualization. (TIF) [file pcbi.1003565.s002.tif]

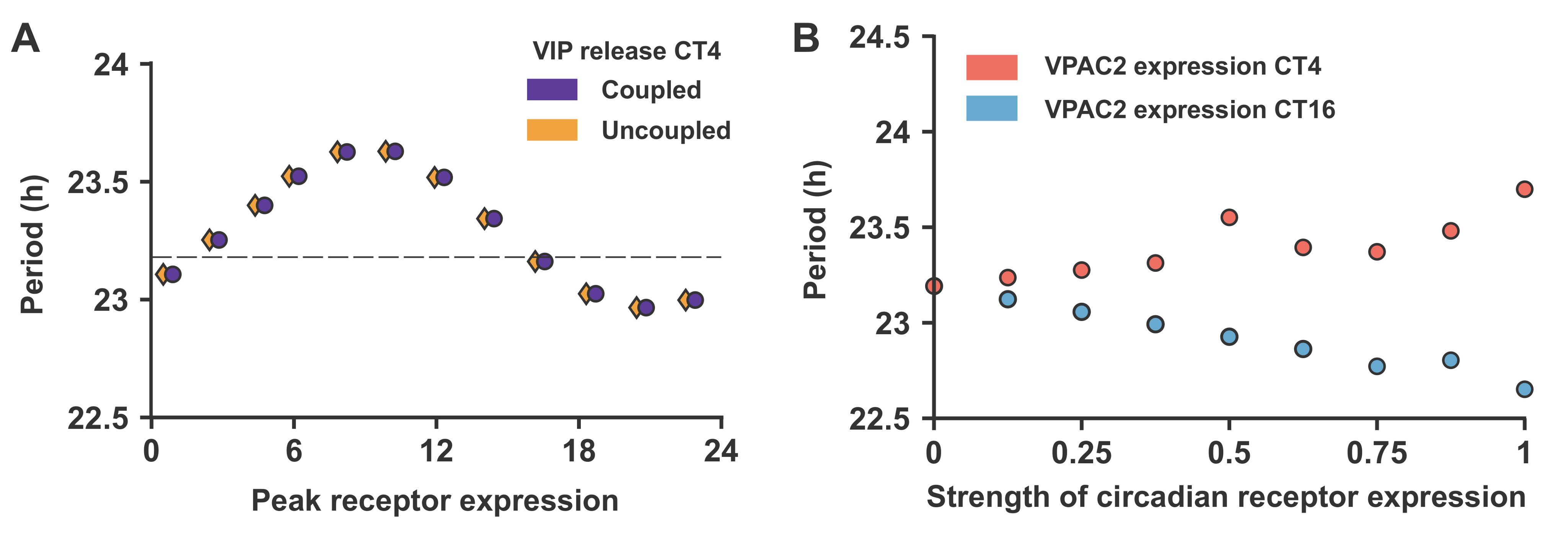

Supplement: Figure S3 — Additional plots to circadian receptor expression simulations in Figure 2 . (A) Modulation of the network period by the timing of VPAC2R expression with VIP release fixed at CT4; (B) Change in the extent of modulation of the network period by the strength of circadian receptor expression with VIP release at CT4. (TIF) [file pcbi.1003565.s003.tif]

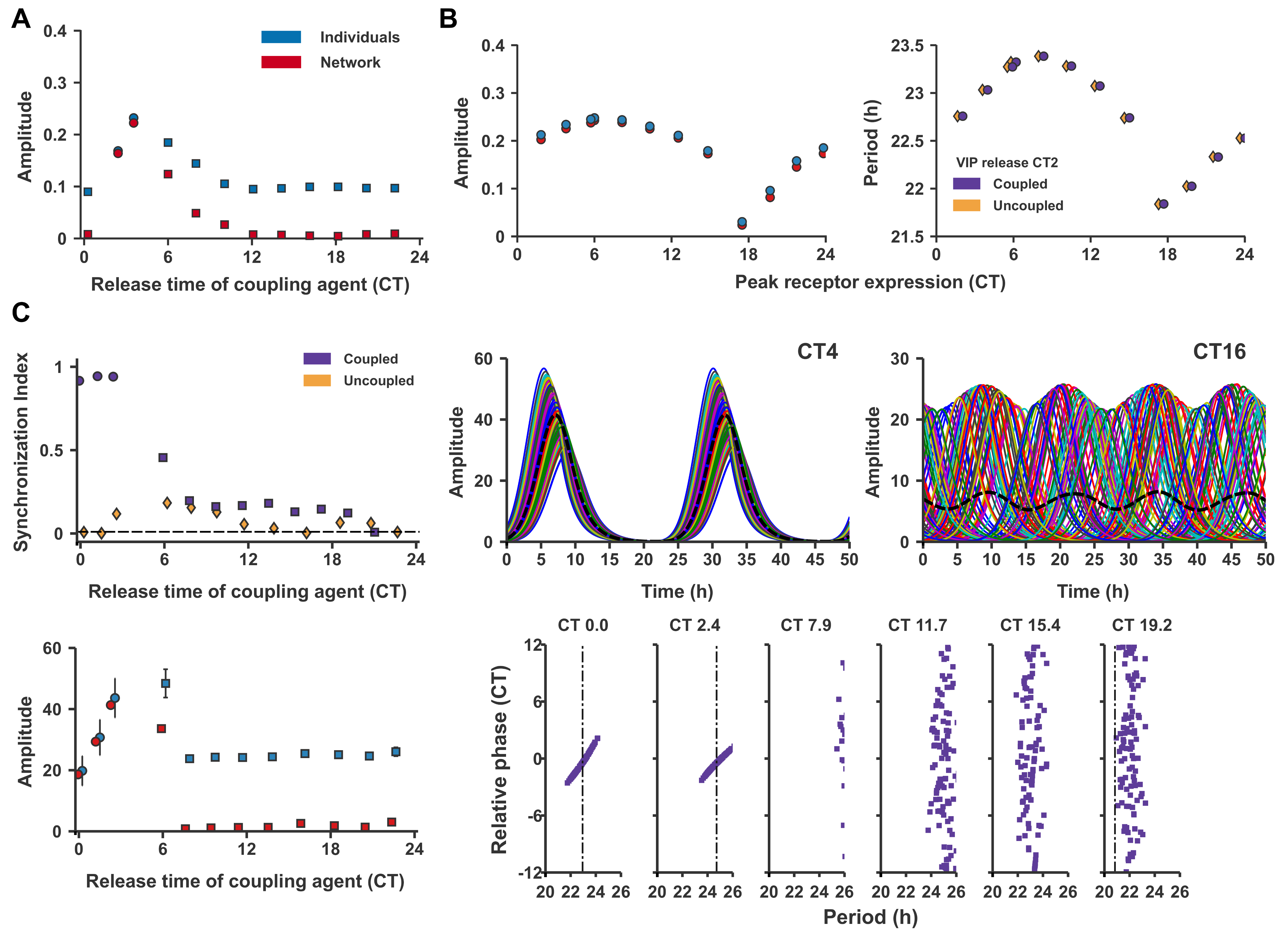

Supplement: Figure S4 — Additional plots to simulations in Figure 3 . (A) Comparison of network and mean individual neuron amplitudes for the Gonze et al. model [26] for the core oscillator (see (7)) for different phases for VIP release; (B) Modulation of the network amplitude (left) and period (right) by the timing of VPAC2R expression with VIP release fixed at CT2 in the Gonze et al. model; (C) Effect of phase of VIP release in the DDE model in this work for multiplicative (‘AND’ logic gate) activation of Per transcription by VIP-VPAC2R coupling as defined in (5). As in Figure 1, the synchronization index, the network time-courses at two different VIP release phases, comparison of network and individual neuron amplitudes and the phase ordering of neurons within the network are shown. (TIF) [file pcbi.1003565.s004.tif]
